# Combining targeted instrument-assisted soft tissue mobilization applications and neuromuscular exercises can correct forward head posture and improve the functionality of patients with mechanical neck pain: a randomized control study

**DOI:** 10.1186/s12891-021-04080-4

**Published:** 2021-02-21

**Authors:** Konstantinos Mylonas, Pavlos Angelopoulos, Evdokia Billis, Elias Tsepis, Konstantinos Fousekis

**Affiliations:** Human Evaluation and Rehabilitation Laboratory, Physical Therapy Department, School of Health Rehabilitation Sciences, University of Patras, Psaron 6, 25100 Egio, Greece

**Keywords:** Forward head posture, IASTM, Neck pain

## Abstract

**Background:**

The purpose of this study was to evaluate the short- and intermediate-term effects of the combined application of instrument-assisted soft tissue mobilization (IASTM) techniques and neuromuscular exercises of the cervical and thoracic area on the improvement of the functionality of patients with mechanical neck pain and accompanying forward head posture (FHP).

**Methods:**

Twenty patients with neck pain and FHP were randomized and received eight treatment sessions of either targeted IASTM in combination with neuromuscular exercises (Group A) or a classical massage and the same set of exercises (Group B). The cervical vertebral angle (CVA), cervical range of motion (ROM) and strength, pain (visual analogue scale-VAS), and neck disability index (NDI) were measured throughout the treatment period and in the two- and four-week post-treatment periods.

**Results:**

The combined application of IASTM and neuromuscular exercises contributed to a significantly greater improvement in CVA (Group A: + 7,2 deg vs Group B: + 1,1 deg) and NDI (Group A:-25,2 vs Group B:-5,8) than massage and the application of the same exercises. Both interventions improved cervical ROM and strength in the short term. Pain was also significantly improved in both groups in both the short (Group A VAS: − 5,97 vs Group B VAS: − 3,1) and intermediate term (Group A VAS:-5,5 vs Group B:-1,5).

**Conclusions:**

Combining IASTM and exercises for the cervical and thoracic area can induce positive postural adaptations and improve the functional status of neck pain patients.

**Trial registration:**

ISRCTN, ISRCTN54231174. Registered 19 March 2020 - Retrospectively registered.

## Background

Cervical pain syndrome is a pathological manifestation with a high epidemiological incidence rate [1, 2] resulting from many aetiological factors. These factors include muscle strains or ligament sprains, pathological adaptations of the cervical soft tissues and poor posture [3]. Of the above aetiological factors, postural pathological adaptations of the human body are particularly associated with the creation of stress and pain conditions in the cervical region. Epidemiological studies have shown that poor posture and neck control occur almost entirely from puberty, with forward head posture (FHP) recorded as the most common orthostatic deviation of the neck [4, 5].

Several treatment techniques and methods are used to rehabilitate pathologies of the cervical spine, including manual therapy, massage, stretching, soft-tissue techniques, and therapeutic exercise [5, 6]. Manual therapy includes hands-on therapy techniques, such as soft tissue mobilization and massage techniques, as well as techniques using therapeutic equipment, such as stainless-steel tools, that allow clinical therapists to identify and treat soft tissue dysfunctions [7, 8]. Therapeutic exercise in the form of neuromuscular retraining is also one of the most important therapeutic interventions for the treatment of cervical pain, as it can improve the mobility of structures, increase muscle strength and ligament tensile strength and prevent tendon injuries [9, 10].

Despite the above-mentioned positive physical adaptations observed after the application of soft tissue techniques and therapeutic exercise, to date, no research has evaluated the effort to correct the overall posture of the human body using these two therapeutic approaches. This scientific deficit is particularly important considering that pathological posture syndromes, such as FHP, are accompanied or caused by other pathological adjustments of the body, such as rounded shoulders, chest kyphosis, and anterior pelvis shift [11, 12].

In the context of this research deficit, the objective of this research is to comparatively evaluate the short- and intermediate-term effects of a possible postural correction of the body in patients with cervical syndrome and coexisting pathological physical adaptations. In particular, the main objective of this research is to assess the effectiveness of the combined application of soft tissue techniques of the cervical and thoracic spine and a therapeutic exercise program for neuromuscular strengthening of specific anatomical areas in correcting the posture and functionality of patients with mechanical neck pain syndrome and accompanying FHP.

## Methods

### Participants

The research sample consisted of 20 female adult patients, aged 43–65 years, weighing 51–73 kg with a height of 1.56–1.75 m, with a diagnosis of mechanical neck pain syndrome and accompanying pathological adjustments of the body, such as FHP. All patients were informed of the objectives of the research and subsequently provided written consent for voluntary participation in the measurements. The study adhered to the CONSORT guidelines and was approved by the Institutional Review Board of the Physical Therapy Department at the University of Patras. For mixed ANOVA, by using G-power software [13] and based on the study design (number of groups = 2, number of measurements = 5, correlation among repeated measures = 0.5, non-sphericity correction epsilon = 1, error type I = 0.05, effect size = 0.27, partial η2 = 0.07, power = 0.8), the minimum sample size was estimated to be 18. The inclusion criteria included female patients with a diagnosis of mechanical neck pain syndrome (cervical soft tissue pathologies, cervical strain/sprain or myofascial pain) from a medical orthopaedic doctor and pain symptoms lasting over 3 months accompanied by FHP based on a cranioverterbral angle (CVA) of < 50°. The selection of a CVA < 50° as the reference angle for the presence of FHP was based on the study of Yip et al. [14], which reported 55.02° ± 2.86° as the normal range. The evaluation of only female patients was based on the prevalence of the disease, which is higher in women than men [1], to ensure the homogeneity of the sample. The exclusion criteria consisted of patients with little or no anterior head projection (CVA < 50°); patients with minor neck injuries, intervertebral disc hernias, spondylolisthesis, accompanying neurological, musculoskeletal and mental problems; and patients using medication.

The patients were randomly divided by a third party into two groups using an online random generator (https://www.randomizer.org/), receiving either targeted IASTM techniques and neuromuscular exercises (Group A, *N* = 10) or the same exercise prescription accompanied by a classical massage (Group B-control, *N* = 10). The variables evaluated in this study were FHP, cervical ROM and strength, and pain and disability [15]. The evaluation of FHP was based on measuring the CVA according to the procedure proposed by Ruivo et al. [16] and Van Niekerk et al. [17]. Based on these approaches, reflective markers were placed on the tragus of the ear and the spinous process of the seventh cervical vertebra (C7), and two photos were taken before and after the interventions in both upright and sitting positions. The CVA was then calculated by processing the pictures with the IMAGE J computational program (LOCI, University of Wisconsin, USA). This procedure can provide valid and reliable postural indicators in both sitting and standing evaluation [17, 18].

Cervical ROM and strength were assessed with an inclinometer (baseline inclinometer® bubble inclinometer) and a MicroFET2 dynamometer (Hoggan Scientific LLC, Salt Lake City, USA), respectively. Cervical flexion and extension and lateral flexion ROM were assessed in a sitting position, while cervical rotation ROM was assessed in the supine position according to the procedures proposed by Norkin & White [19]. Three measurements per movement were performed, and the mean was used in the analysis. Cervical strength evaluation was performed in the supine position according to the procedure proposed by Tierney et al., 2005. The measurements had to cause no pain, and the research protocol included 3 isometric contractions for 3 s with a 30 s rest between contractions. After familiarization with the measurement, the mean value from the 3 maximal contractions was used in the analysis.

The Visual Analogue scale (VAS) was used for subjective pain assessment, and the neck disability index (NDI) questionnaire was used to record patients’ functional status. A total of eight treatment sessions were performed on all patients, two each week. FHP, ROM, and cervical strength were evaluated before and after each session, while the functionality of the cervical spine through the NDI questionnaire was evaluated five times (before the 1st, 4th, and 8th treatment sessions and at 2 and 4 weeks post-treatment). The therapeutic sessions and evaluations of the participants were carried out in the Laboratory of Human Evaluation and Rehabilitation of the University of Patras. The study outcomes and possible adverse effects from the therapeutic interventions’ application were evaluated by experienced physical therapists who were blind both to the study scope and treatment allocation.

### Therapeutic interventions

Participants in Group A received soft tissue techniques in the form of the ERGON IASTM technique [20] in targeted cervical and thoracic spine areas with the aim of myofascial release of shortened structures. Participants in Group B, for the same purpose, received a classical massage in the same areas. The anatomical areas that received the treatment and detailed data (strokes, treatment direction, speed and duration) of applying the two therapeutic interventions are presented in Table 1. Subsequently, participants in both groups underwent specialized neuromuscular exercises to correct FHP. The duration of each treatment session was 50 min for both research groups. At the beginning of the procedure, the therapist performed a warm-up massage for both groups. In Group A, the massage lasted 10 min and was followed by IASTM application for another 10 min, while in Group B, the massage lasted 20 min. Thus, the overall soft tissue interventions for both groups lasted 20 min.

**Table 1:** Therapeutic interventions of the study

	IASTM	MASSAGE
**Goal**	Myofascial release -Improved tissue elasticity	
**Provider**	ERGON IASTM certified Physical Therapists	Physical Therapists
**Materials**	ERGON IASTM Tools Emollient	Therapists hands Massage emollient
**Procedures**	• General treatment of the anatomical structures of the cervical area, the thorax (back and front) and the shoulder girdle • Personalized treatment aiming at localised points of myofascial restrictions with the aim of myofascial release and tissue relaxation.	
**Treatment Strokes**	ERGON IASTM applications (linear, semi-circular and circular IASTM strokes)	Classical massage strokes (effleurage, petrissage - kneading, friction)
**Direction (s) and location(s) of treatment interventions applications**	Cranial direction: Suboccipital muscles, anterior deltoid, sternocleidomastoid, scalenes. Caudal direction: Cervical extensor and rotator muscles, erector spinae. Lateral direction (toward the spine): Trapezius, scapular muscles, posterior deltoid, pectoralis major Lateral direction (away from the spine): Pectoralis major	
**Speed of applications**	Slow Speed	
**No of intervention sessions/duration.**	Treatments sessions/10 min duration	

**Fig. 1:**
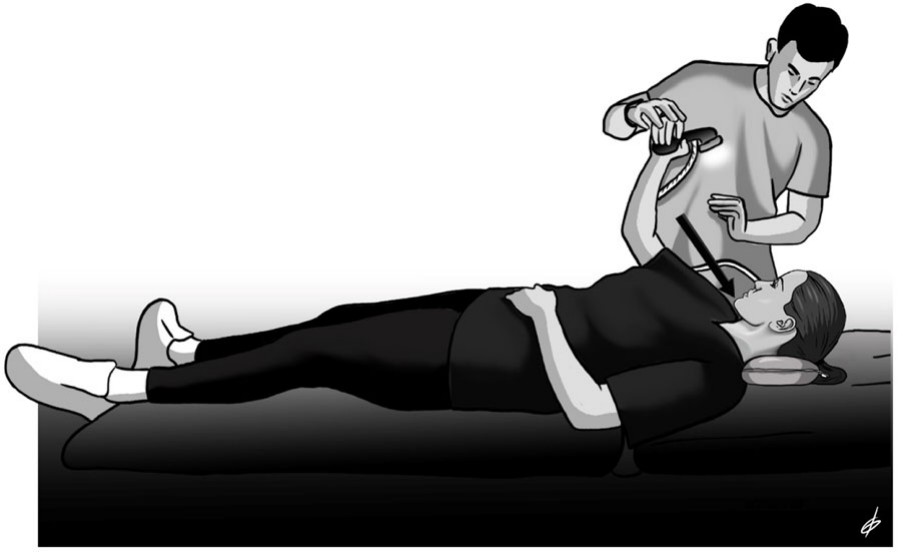
Strengthening of the deep neck flexors with a combination of a neck curl with a chin tuck

**Fig. 2:**
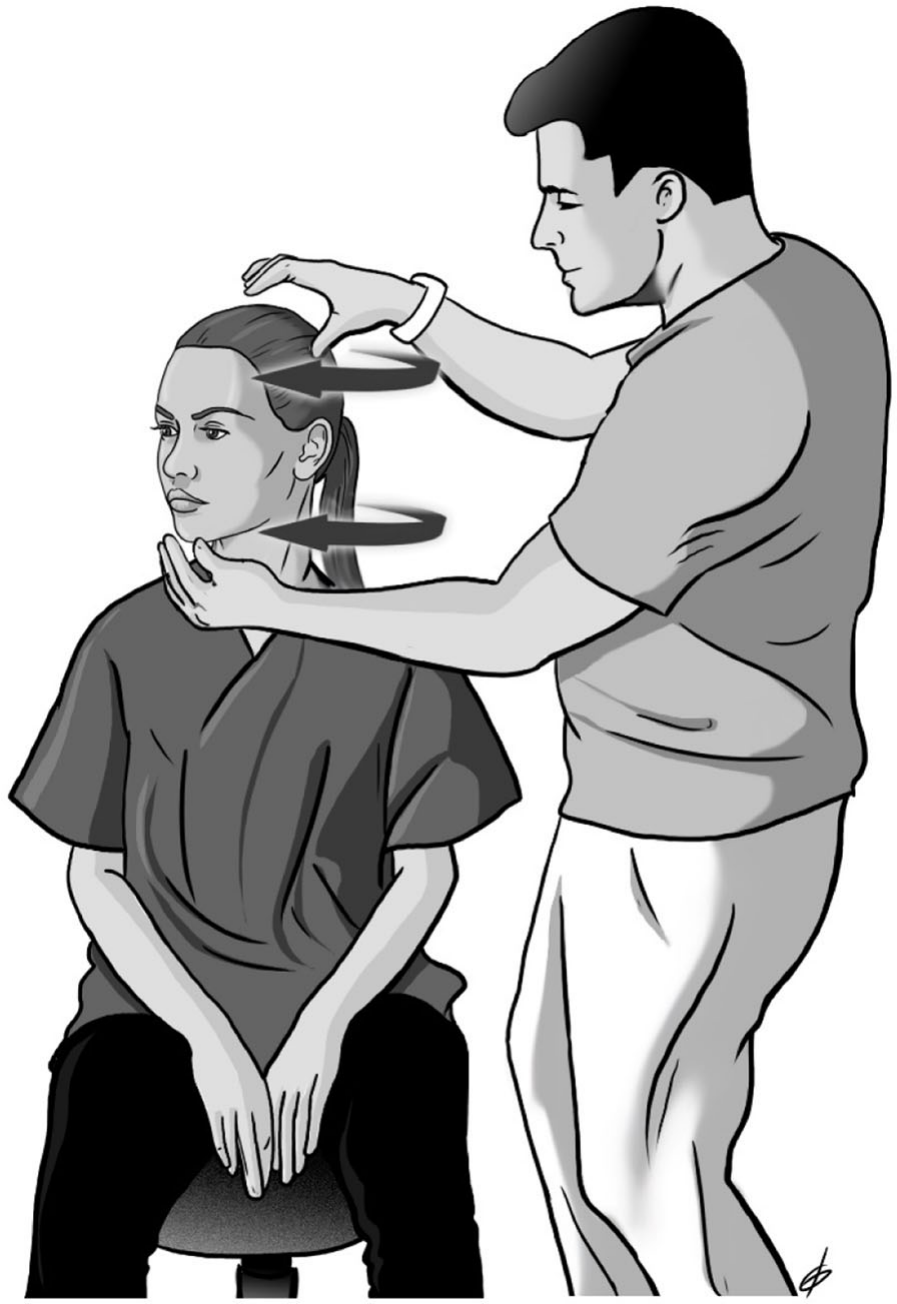
Cervical rotation strengthening through contraction of the deep neck flexors at the same time as the rotating muscles

**Fig. 3:**
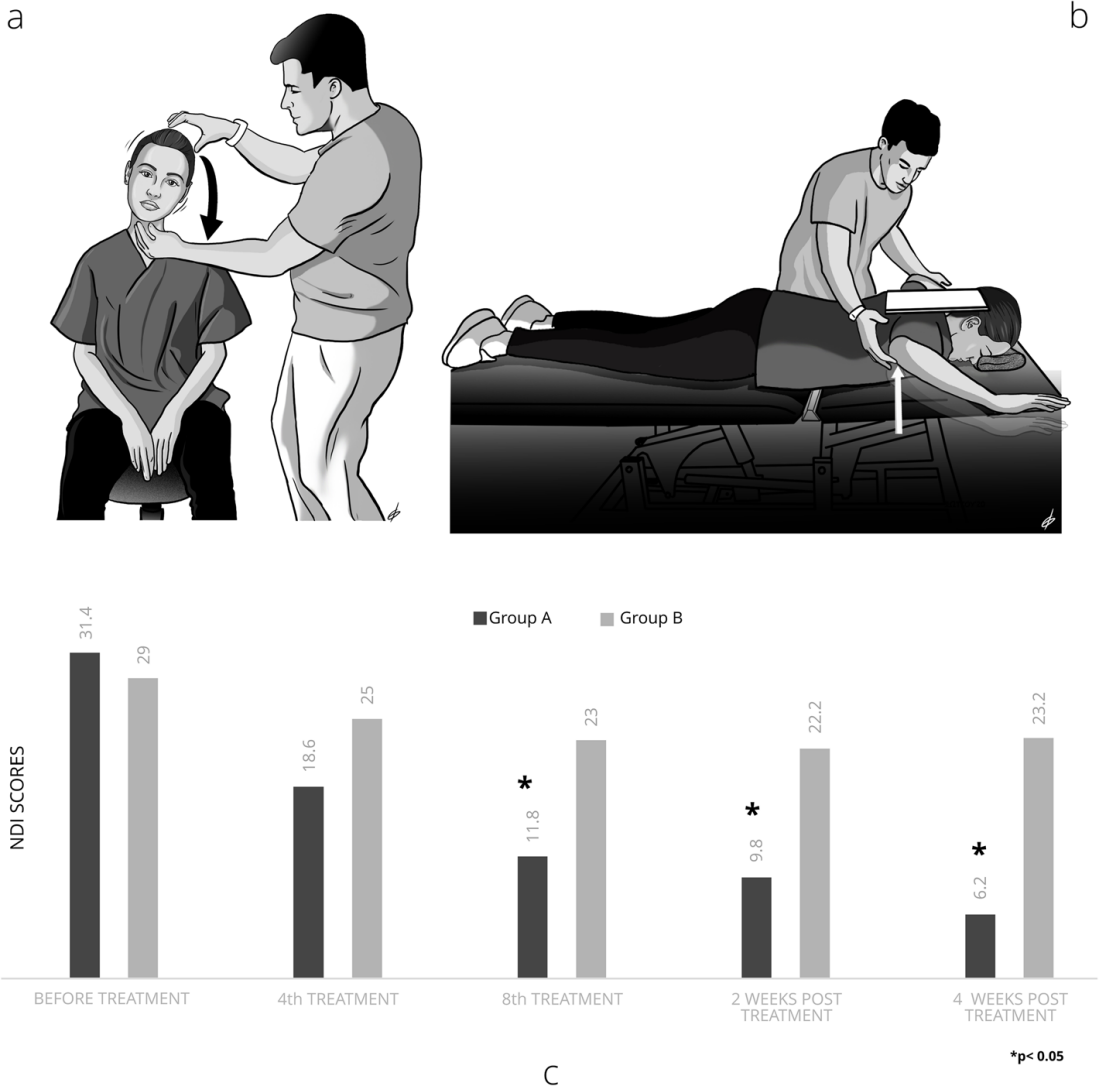
**a** Cervical lateral flexion strengthening through the contraction of the deep neck flexors at the same time as the lateral flexor muscles, **b** Strengthening of the trapezius and rhomboid muscles from the prone position through horizontal abduction of the shoulder blades, **c** NDI score variations according to the treatment interventions and periods

Immediately after the application of the soft tissue techniques, four selected neuromuscular exercises were applied to both groups (Table 2). The first exercise included strengthening of the deep neck flexors with a combination of a neck curl with a chin tuck position in the supine position using the Chattanooga Stabilizer Pressure biofeedback device (Fig. 1) [9]. The second and third exercises included cervical rotation and lateral flexion strengthening through contraction of the deep neck flexors at the same time as the rotating or lateral flexor muscles in a sitting position (Figs. 2 and 3a). Finally, the fourth exercise was aimed at correcting the forward position of the shoulder blades by activating the trapezius and rhomboid muscles from the prone position through horizontal abduction of the shoulder blades (Fig. 3b). The exercises were performed with 10 repetitions and 3 sets, while instructions were given to the patients to perform all the exercises on the other days of the week for the entire 8 weeks of the intervention [21].

**Table 2:** Description of the therapeutic exercises performed during the intervention period

Exercise	Main Muscles	Description
Chin tuck in the supine position	Longus colli Longus capitis	In the supine position, the patient, guided by the Stabilizer Pressure biofeedback device, reaches 5 pressure targets in 2 mmHg increments from a baseline of 20 mmHg until the final level of 30 mmHg by performing a neck curl with the chin tucked. The training starts at the level that the participant can hold steady for 10 s without activation of the superficial neck flexor muscles while performing a slow craniocervical flexion. For each target level, the contraction duration increases to 10 s, and each patient performs 10 repetitions. If three attempts by the patient are successful, then she continues to the next level. The test was stopped when the subject failed to hold for 10 s at the pressure level in any of the 3 repetitions or when she contracted incorrect muscle groups.
Cervical rotation with chin tuck in a sitting position	Longus colli Longus capitis Semispinalis Capitus Semispinalis Cervicis Sternocleidomastoid Longissimus Capitis	In a sitting position, the patient rotates her head in both directions (left-right) with simultaneous contraction of the deep neck flexors (with the chin tucked). The movement stops at the point where the patient leaves the axis of rotation and performs both rotation and lateral flexion.
Cervical lateral flexion with chin tuck in a sitting position	Longus colli Longus capitis Rectus capitis lateralis Scalenes Sternocleidomastoid Obliquus Capitus Superior	In a sitting position, the patient laterally flexes the head in both directions (left-right) with simultaneous contraction of the deep neck flexors (with chin tucked). The movement stops at the point where the patient leaves the axis of movement and performs both rotation and lateral flexion.
Shoulder horizontal abduction with external rotation in the prone position	Middle trapezius Lower trapezius Rhomboids Infraspinatus Teres minor	In the prone position, the patient horizontally abducts and externally rotates the shoulders with the elbow flexed at 90°. The patient lifts both shoulders at the same time, trying to squeeze both scapulae together, avoiding movement of the head, which rests in the neutral position.

### Statistical analysis

To compare the effectiveness of the intervention programs, as well as to investigate their effects over time, the mixed ANOVA method was used with univariate analysis. For statistical analysis of the data, the statistical software SPSS-25 was used. The minimum value of the statistical significance level, the *p*-value, in all the statistical tests was set at 5%.

## Results

The participants’ functional data before and after the therapeutic interventions are displayed in Table 3. The application of the study’s therapeutic interventions to the participants did not lead to any adverse events. The results showed significant improvement in the mean FHP while sitting, and was higher in Group A, which received IASTM and corrective exercises, than in Group B, which received a massage and the same exercises. The improvement of Group A was maintained both at two (*p* = 0.397) and 4 weeks (*p* = 0.080) post-treatment. In Group A, there was a statistically significant improvement in FHP in the upright position immediately after the last treatment (*p* = 0.0005), which was not maintained two (*p* = 0.01) or 4 weeks (*p* = 0.004) post-treatment.

**Table 3:** Mean values for FHP and cervical pain, ROM, strength and disability before and after therapeutic interventions in patients with mechanical neck pain (*N* = 20)

Variables		Treatment	1st Treatment		4th Treatment		8th Treatment		2 weeks post-treatment		4 weeks post-treatment	
			Group A	Group B	Group A	Group B	Group A	Group B	Group A	Group B	Group A	Group B
			Mean (sd)	Mean (sd)	Mean (sd)	Mean (sd)	Mean (sd)	Mean (sd)	Mean (sd)	Mean (sd)	Mean (sd)	Mean (sd)
Forward head position (deg)	CVA in sitting	Before	41,4 (3,2)	42,4 (3,2)	43,7 (3,1)	43,7 (3,6)	46,4 (2,7)	44,6 (3,20)	49,2 (2,6)	44,2 (3,3)	48,6 (2,4)	43,5 (3,0)
		After	45,7 (2,8)	43,4 (2,9)	47,3 (3,6)	45,0 (2,8)	49,9 (2,7)	46,0 (3,0)				
	CVA in standing	Before	46,9 (1,6)	47,0 (2,8)	48,7 (2,0)	47,2 (2,3)	50,8 (1,6)	48,7 (2,4)	52,6 (2,0)	48,5 (2,4)	52,0 (1,9)	47,1 (2,6)
		After	50,3 (1,7)	48,0 (2,2)	52,1 (2,1)	48,9 (2,0)	54,1 (1,5)	50,5 (2,1)				
Pain	VAS scale	Before	6,1 (1,3)	4,0 (2,3)	3,1 (1,8)	2,8 (2,0)	0,4 (0,3)	1,5 (1,4)	0,4 (0,5)	2,0 (1,5)	0,6 (0,5)	2,5 (1,6)
		After	3,6 (1,5)	3,4 (2,1)	1,6 (1,4)	1,9 (1,3)	0,2 (0,4)	0,9 (0,9)				
Cervical range of motion (deg)	Flexion	Before	58,7 (8,5)	54,5 (9,8)	64,2 (12,7)	54,4 (12,2)	69,7 (9,4)	58,2 (11,0)	70,2 (7,9)	55,8 (8,9)	69,0 (6,1)	55,3 (8,6)
		After	64,7 (6,6)	58,1 (9,6)	70,1 (9,7)	61,4 (8,6)	71,4 (9,2)	62,9 (12,3)				
	extension	Before	54,5 (12,7)	53,0 (8,8)	59,6 (9,2)	57,2 (9,8)	63,5 (10,7)	61,1 (11,9)	65,2 (8,6)	61,5 (10,3)	65,4 (9,3)	60,4 (11,4)
		After	60,6 (11,8)	52,9 (11,3)	68,7 (13,5)	61,4 (10,10)	67,5 (9,4)	59,5 (8,9)				
	Lateral flexion (right)	Before	42,8 (7,9)	36,0 (8,7)	49,7 (9,3)	40,0 (14,3)	55,0 (7,8)	46,2 (15,2)	58,1 (10,4)	45,1 (12,9)	58,5 (8,9)	43,0 (14,5)
		After	46,5 (5,8)	43,6 (11,5)	56,2 (9,42)	43,8 (11,5)	62,3 (8,6)	49,4 (11,5)				
	Lateral flexion (left)	Before	43,0 (6,3)	37,0 (4,8)	48,3 (9,6)	41,8 (11,7)	57,3 (9,6)	47,0 (12,9)	57,1 (9,6)	46,4 (12,5)	58,4 (9,9)	46,7 (13,2)
		After	47,8 (6,2)	44,5 (9,6)	56,5 (9,8)	45,5 (12,3)	64,6 (9,4)	51,7 (9,6)				
	Rotation (right)	Before	62,5 (17,0)	61,5 (17,8)	73,4 (16,4)	62,3 (17,1)	75,2 (6,3)	65,1 (18,9)	80,3 (7,8)	66,4 (20,6)	75,6 (11,8)	63,2 (17,9)
		After	76,0 (14,2)	57,8 (16,3)	78,5 (10,92)	66,1 (18,4)	83,0 (5,9)	70,4 (20,7)				
	rotation (left)	Before	64,5 (14,1)	62,4 (17,5)	70,5 (13,5)	64,6 (16,3)	76,6 (7,3)	66,3 (16,9)	79,9 (5,0)	69,3 (18,7)	79,0 (4,9)	64,0 (16,9)
		After	76,5 (15,1)	61,9 (17,6)	78,8 (13,4)	68,6 (17,5)	84,0 (6,1)	67,8 (17,7)				
Strength (kgr)	Flexion	Before	24,8 (13,2)	16,4 (3,9)	26,3 (8,8)	18,2 (7,0)	33,3 (10,5)	27,0 (9,9)	34,5 (9,7)	27,7 (10,6)	37,1 (12,5)	28,0 (10,5)
		After	30,0 (8,4)	19,7 (7,2)	32,1 (11,9)	22,2 (8,7)	40,3 (12,0)	29,2 (12,2)				
	Extension	Before	83,6 (22,3)	77,6 (30,8)	95,1 (35,1)	89,6 (44,3)	109,1 (34,6)	99,3 (51,7)	108,0 (35,4)	99,7 (50,4)	111,3 (31,1)	95,0 (45,5)
		After	90,4 (20,7)	76,5 (32,7)	103,0 (32,6)	92,2 (50,5)	127,1 (28,4)	104,1 (51,8)				
	Lateral flexion (right)	Before	35,1 (20,7)	30,4 (10,5)	42,1 (17,1)	35,5 (10,7)	44,0 (17,1)	48,3 (20,8)	48,0 (16,6)	46,9 (19,2)	51,0 (14,7)	47,1 (18,4)
		After	44,3 (23,2)	30,3 (12,2)	45,3 (23,2)	38,9 (11,5)	50,3 (16,8)	55,8 (25,9)				
	Lateral flexion (left)	Before	39,3 (29,3)	29,5 (9,6)	39,7 (25,5)	35,1 (14,7)	47,7 (23,4)	47,1 (26,2)	47,7 (17,4)	43,6 (17,5)	50,8 (13,3)	43,4 (17,8)
		After	46,3 (26,6)	32,6 (12,7)	46,5 (26,2)	41,7 (14,1)	54,0 (22,3)	52,6 (28,9)				
Disability	Neck disability Index	before	31,4 (10,1)	29 (11,1)	18,6 (6,7)	25 (10,8)	11,8 (3,9)	23 (10,5)	9,8 (3,6)	22,2 (9,2)	6,2 (1,9)	23,2 (7,2)

A statistically significant increase in the mean ROM of cervical flexion and extension was found immediately after the last treatment in both groups (*p* = 0.002 and *p* = 0.0005, respectively). Furthermore, the average ROM values were maintained after two (*p* = 0.151 and *p* = 1.000, respectively) and 4 weeks (*p* = 0.064 and *p* = 1.000, respectively) in both groups. Cervical flexion strength also increased immediately after the last treatment (*p* = 0.017) in Group A, and this increase was greater to that found in Group B. This improvement was not maintained 2 weeks post-treatment (*p* = 0.019); however, after 4 weeks, the improvement in strength was restored immediately after the last treatment (*p* = 1.000). No statistically significant differences were found between the two types of therapeutic interventions for any other strength evaluation.

A statistically significant decrease in the mean value of the VAS pain scale was observed in both groups immediately after the last treatment (*p* = 0.0005). This decrease was maintained in both groups after 2 and 4 weeks (*p* = 0.0005 and *p* = 0.008). NDI was also improved in both groups. This improvement was statistically greater in Group A compared to Group B after the eighth treatment session (Group A: mean NDI score = 11.8; Group B: mean NDI score = 22; Z = − 2.864, *p* = 0.004) and after 2 weeks (Group A: mean NDI score = 9.8; Group B: mean NDI score = 22.2; Z = 3.467, *p* = 0.001) and 4 weeks post-treatment (Group A: mean NDI score = 6.2; Group B: mean NDI score = 23.2; Z = − 3.804, *p* = 0.0005, Fig. 3c).

## Discussion

IASTM application in targeted areas of the body combined with neuromuscular corrective exercises improved FHP, ROM strength, and the functionality of women with painful cervical syndrome to a greater extent than a similar program including classical massage techniques instead of IASTM. The improvement of FHP is a very important and innovative finding, as it highlights the possibility of correcting pathological postural adjustments through IASTM techniques in targeted areas and neuromuscular retraining exercises. In other words, it seems that targeted myofascial techniques are more effective than a classical massage to create conditions for myofascial tissue release and form the basis for creating positive postural adjustments when combined with specialized neuromuscular retraining exercises. The above finding is partly supported by the results of Kim et al. [22], who also showed a short-term improvement in FHP when examining the effects of soft tissue techniques and strengthening exercises on the suboccipital muscles.

The cervical ROM and strength of most cervical movements appear to have been positively affected by both therapeutic interventions without significant differences between them. A ROM improvement was found in the short-term for most cervical movements, and this finding is consistent with many studies that have evaluated the short-term effect of IASTM techniques on flexibility. Strength adaptations were slightly different, as cervical flexion strength improved significantly immediately after the last treatment in the group that received combined IASTM soft tissue techniques and neuromuscular exercises, but it decreased 2 weeks after the last treatment. However, 4 weeks after completion of the main treatment, there was an increase in cervical flexion strength back to the level of its initial improvement. The above findings on the positive strength adjustments after the targeted strength training programs are strongly supported by several studies that concluded that four- to six-week strength training programs can lead to muscle hypertrophy and increased strength in specific muscle groups [23, 24]. It can also be assumed that the improvement in the anterior displacement of the head observed in these patients contributed to the formation of positions of better biomechanical function and a mechanical advantage of the cervical muscles that contribute to cervical flexion, leading to an intermediate-term improvement in strength production. Based on the fact that force is transmitted through the connective tissue in and around muscle and in non-muscular connective tissues [25], it can be assumed that soft-tissue techniques with special equipment enhanced the myodynamic adaptations by improving the mobility of the connective tissue in general. The maintenance of the strength improvement after completion of the main intervention and for a period of 4 weeks can be explained by the fact that patients, after completing the basic treatment lasting 4 weeks, continued the neuromuscular retraining exercises at home. The findings of this study confirm older findings that have shown a short-term improvement in function and FHP through systematic and targeted strengthening of neck muscles [26]. In contrast, Wright et al. [27] reported that strengthening the neck muscles does not improve FHP or the functionality of the specific anatomical area. However, this study did not combine soft tissue and strengthening techniques, and the strengthening program lasted only 1 month.

Both therapeutic interventions significantly reduced patient pain immediately after the last treatment, and this improvement was maintained and strengthened 2 and 4 weeks after the end of the last treatment, without significant differences between groups. The above results are in agreement with findings from other studies on the short-term improvement of pain symptoms with the use of myofascial release programs and therapeutic exercise programs to treat myofascial painful syndromes [28, 29]. The maintenance of the low levels of pain observed in this study cannot be compared with similar studies, as no corresponding studies have used the methodological design of the present study and no similar studies have evaluated the overall functional capacity and disability of patients in the long term. However, it can be assumed that the improvement of FHP, as well as of the functional capacity expressed by the improvement of ROM and strength observed in the study patients and in combination with home-based exercise, is directly related to the reduction of pain symptoms in patients with mechanical neck pain. The above theoretical conclusion is reinforced by the findings of Yip et al. [14], who stated that forward head posture is one of the factors related to neck pain and disabilities in patients with neck pain.

Patient disability was also improved in both intervention groups over the course of this research. However, this improvement was statistically more significant in Group A, which received IASTM therapy, than in Group B. Although patients in both groups started with the same level of cervical disability, the mean cervical disability level of Group A was significantly lower than that of Group B, which received a classical massage both in the short- and long-term. This significant functional adaptation cannot be supported or confirmed by similar findings because no studies have systematically evaluated the improvement in FHP and functional status of patients with cervical pain. This significant reduction in patient disability can be attributed to the biomechanical correction of FHP that was greater in Group A than in Group B, and to the better myodynamic adaptations observed in this group. In particular, the almost 5-degree improvement in CVA observed between the two groups at the end of the treatment can reduce postural stress and ultimately improve neck pain patients’ functionality by improving cervical, thoracic and scapular kinematics and muscle activity.

The findings of this study should be evaluated with consideration of its limitations. Specifically, the patients evaluated in the present study were not recruited by random sampling but were a convenience sample from the same geographical area (Attica-Greece). Additionally, although the participants showed relative homogeneity in their basic physiological characteristics, there was no homogeneity or reference to the pathology that led to cervical pain. Thus, the study included as many patients as possible with cervical mechanical pain. It is well known, however, that mechanical pain is the result of multifactorial aetiologies and of many different pathologies that can range from cervical muscle strain to facets and ligament restrictions. Additionally, there were differences in patients’ physical conditions, as some of the patients had never participated in exercise programs in the past.

## Conclusions

IASTM techniques, combined with neuromuscular retraining exercises based on a holistic model of treatment of the human body, can significantly reduce pain and improve the corresponding function of patients with cervical pain compared to the application of the same exercises and a simple massage. These results need to be supported with future studies that include larger samples and also target lumbar postural dysfunctions.

## Data Availability

The datasets used and/or analysed during the current study are available from the corresponding author upon reasonable request.

## Abbreviations

IASTM: Instrument-assisted soft tissue mobilization
FHP: Forward head posture
ROM: Range of motion
NDI: Neck disability index
CVA: Cervical vertebral angle
