# Correction to: Combining targeted instrument-assisted soft tissue mobilization applications and neuromuscular exercises can correct forward head posture and improve the functionality of patients with mechanical neck pain: a randomized control study

**DOI:** 10.1186/s12891-021-04243-3

**Published:** 2021-04-26

**Authors:** Konstantinos Mylonas, Pavlos Angelopoulos, Evdokia Billis, Elias Tsepis, Konstantinos Fousekis

**Affiliations:** Human Evaluation and Rehabilitation Laboratory, Physical Therapy Department, School of Health Rehabilitation Sciences, University of Patras, Psaron 6, 25100 Egio, Greece

**Correction to: BMC Musculoskelet Disord 22, 212 (2021)**

**https://doi.org/10.1186/s12891-021-04080-4**

Following publication of this article [1], the authors acknowledge the following Correction to the Funding Section:

“The Ergon® IASTM technique was created at the Department of Physiotherapy of the University of Patras, where it is taught both in undergraduate and postgraduate level as well as in external educational programs. The authors KM, AP, and KF have participated in developing and disseminating this specific program. In the present study, the authors KM, AP, and KF participated exclusively in designing this study and methods, and they were not involved in the application of therapeutic interventions, measurements, and/or data handling”.
